# Supplementary material for: The Na+/K+-ATPase generically enables deterministic bursting in class I neurons by shearing the spike-onset bifurcation structure
Source: PLoS Comput Biol. 2024 Aug 12;20(8):e1011751. doi: 10.1371/journal.pcbi.1011751 (PMC11383233; doi:10.1371/journal.pcbi.1011751)
Supplement: S1 Fig — In the main manuscript, the extracellular potassium concentration ([K+]out) is a variable of special interest, and for consistency, all bifurcations and slow subsystem dynamics are depicted with [K+]out on the y-axis. Here, we present the dynamic analysis of the burst (Figs 3B and 4) in the more conventional way (with the independent variable [K+]out on the x-axis). Additionally, we include a more complete depiction of the fast subsystem bifurcation. (A) A zoom out of Fig 3B is illustrated here. The bistable region between the saddle-node (SN) and homoclinic (HOM) bifurcation is the bursting region (indicated by the dotted rectangular). Note that there is another bistable region between HOM and Hopf bifurcations where a stable node and a stable limit cycle coexist. According to the slow subsystem dynamics, [K+]out increases if the fast subsystem spikes. On the stable branch of the fast subsystem emerging from SN bifurcation, [K+]out decreases. From the Hopf bifurcation of the fast subsystem, another stable node is added to the fast subsystem. If the fast subsystem is on this stable node, the complete system dynamics dictates an increase of the [K+]out. (B) Phase portrait of the complete burst (as a function of [K+]out horizontally and voltage vertically, black line giving the trajectory) overlaid with the one-parameter ([K+]out) bifurcation diagram of the fast subsystem (coloured symbols and lines). The bursting trajectory is identical to that in Fig 2A; the diagram corresponds to Fig 3B with flipped axes. Additionally, Vmin of the stable limit cycle (lower green line) and the unstable focus (dashed purple line) are depicted. (C) Hysteresis loop of the slow subsystem overlaid with the bifurcations of the fast subsystem. The [K+]out dynamics of the reduced, slow subsystem entails a hysteresis loop oscillator. The slow oscillation organises around the bistable region of the fast subsystem, as also shown in panels A and B. The red and blue lines represent the location [file pcbi.1011751.s001.pdf]

S1 Fig for:

The Na^+/K^+-ATPase generically enables deterministic bursting in class I neurons by shearing the spike-onset bifurcation structure

Mahraz Behbood[1,2], Louisiane Lemaire[1,2], Jan-Hendrik Schleimer[1,2], Susanne Schreiber[1,2, *]

[1] Institute for Theoretical Biology, Department of Biology, Humboldt-Universität zu Berlin, Philippstraße 13, 10115 Berlin, Germany

[2] Bernstein Center for Computational Neuroscience, Philippstr. 13, 10115 Berlin, Germany

[*] Corresponding author E-mail: s.schreiber@hu-berlin.de (SS)

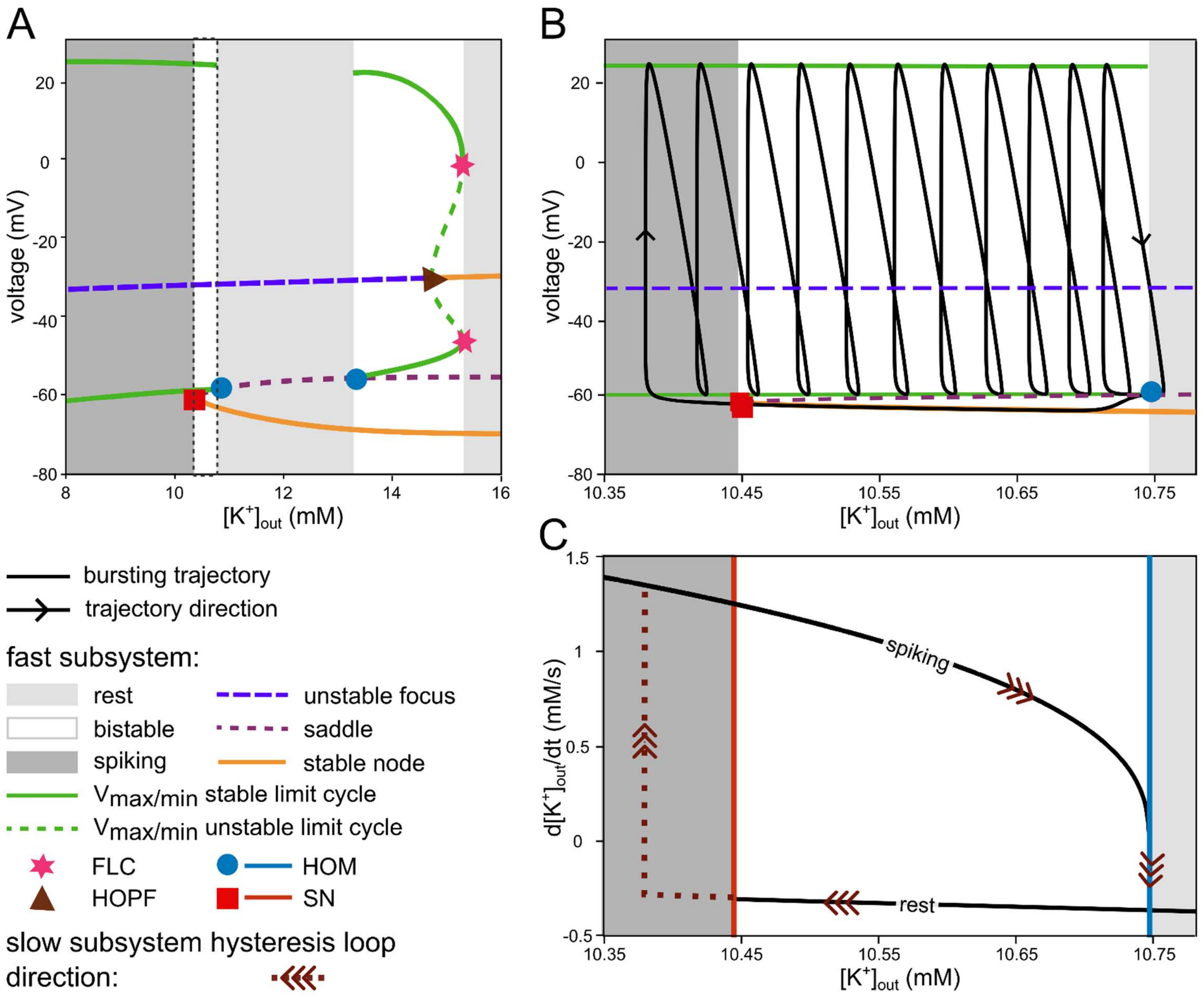

S1 Fig. An overview of the bursting dynamics analysis.

In the main manuscript, the extracellular potassium concentration ($[K^+]_{out}$) is a variable of special interest, and for consistency, all bifurcations and slow subsystem dynamics are depicted with $[K^+]_{out}$ on the y-axis. Here, we present the dynamic analysis of the burst (Figs 3B and 4) in the more conventional way (with the independent variable $[K^+]_{out}$ on

the x-axis). Additionally, we include a more complete depiction of the fast subsystem bifurcation. (**A**) A zoom out of Fig 3B is illustrated here. The bistable region between the saddle-node (SN) and homoclinic (HOM) bifurcation is the bursting region (indicated by the dotted rectangular). Note that there is another bistable region between HOM and Hopf bifurcations where a stable node and a stable limit cycle coexist. According to the slow subsystem dynamics, $[K^+]_{out}$ increases if the fast subsystem spikes. On the stable branch of the fast subsystem emerging from SN bifurcation, [K+]out decreases. From the Hopf bifurcation of the fast subsystem, another stable node is added to the fast subsystem. If the fast subsystem is on this stable node, the complete system dynamics dictates an increase of the $[K^+]_{out}$. (**B**) Phase portrait of the complete burst (as a function of $[K^+]_{out}$ horizontally and voltage vertically, black line giving the trajectory) overlaid with the one-parameter ($[K^+]_{out}$) bifurcation diagram of the fast subsystem (coloured symbols and lines). The bursting trajectory is identical to that in Fig 2A; the diagram corresponds to Fig 3B with flipped axes. Additionally, V_{min} of the stable limit cycle (lower green line) and the unstable focus (dashed purple line) are depicted. (**C**) Hysteresis loop of the slow subsystem overlaid with the bifurcations of the fast subsystem. The $[K^+]_{out}$ dynamics of the reduced, slow subsystem entails a hysteresis loop oscillator. The slow oscillation organises around the bistable region of the fast subsystem, as also shown in panels A and B. The red and blue lines represent the location of the saddle-node (SN) and homoclinic (HOM) bifurcations of the fast subsystem as a function of $[K^+]_{out}$, respectively. This diagram corresponds to Fig 4 with flipped axes. For more information on how to calculate the reduced slow subsystem see Methods (Slow-fast method), and Fig 4 in the main manuscript.
