# Supplementary material for: The Na+/K+-ATPase generically enables deterministic bursting in class I neurons by shearing the spike-onset bifurcation structure
Source: PLoS Comput Biol. 2024 Aug 12;20(8):e1011751. doi: 10.1371/journal.pcbi.1011751 (PMC11383233; doi:10.1371/journal.pcbi.1011751)
Supplement: S2 Fig — The spike-adding mechanism at play in our model appears to involve canard solutions. We show here a jump-on canard, found at the transition between the 11-spike burst that was used as illustrative example throughout this paper (Figs 2–4), and a 12-spike burst obtained when increasing the pump density Imax. Jump-on canards are particular solutions that connect a fast and attractive component (here when the neurons spikes), to a slow and repulsive component of the dynamics (here the short pause in the spiking within the burst), see for example [85,86]. (A) Voltage trace during one period. (B) Potassium trace during one period. (C) Voltage trajectory superimposed onto the bifurcation diagram of the fast subsystem with respect to potassium. This trajectory follows for a while the repelling slow manifold defined by the saddle point of the fast subsystem involved in the homoclinic bifurcation (orange dashed line). Iapp = 0.5 μA/cm2, Imax = 0.99450852625 μA/cm2. (PDF) [file pcbi.1011751.s002.pdf]

S2 Fig for:

The Na^+/K^+-ATPase generically enables deterministic bursting in class I neurons by shearing the spike-onset bifurcation structure

Mahraz Behbood[1,2], Louisiane Lemaire[1,2], Jan-Hendrik Schleimer[1,2], Susanne Schreiber[1,2,*]

[1] Institute for Theoretical Biology, Department of Biology, Humboldt-Universität zu Berlin, Philippstraße 13, 10115 Berlin, Germany

[2] Bernstein Center for Computational Neuroscience, Philippstr. 13, 10115 Berlin, Germany

[*] Corresponding author E-mail: s.schreiber@hu-berlin.de (SS)

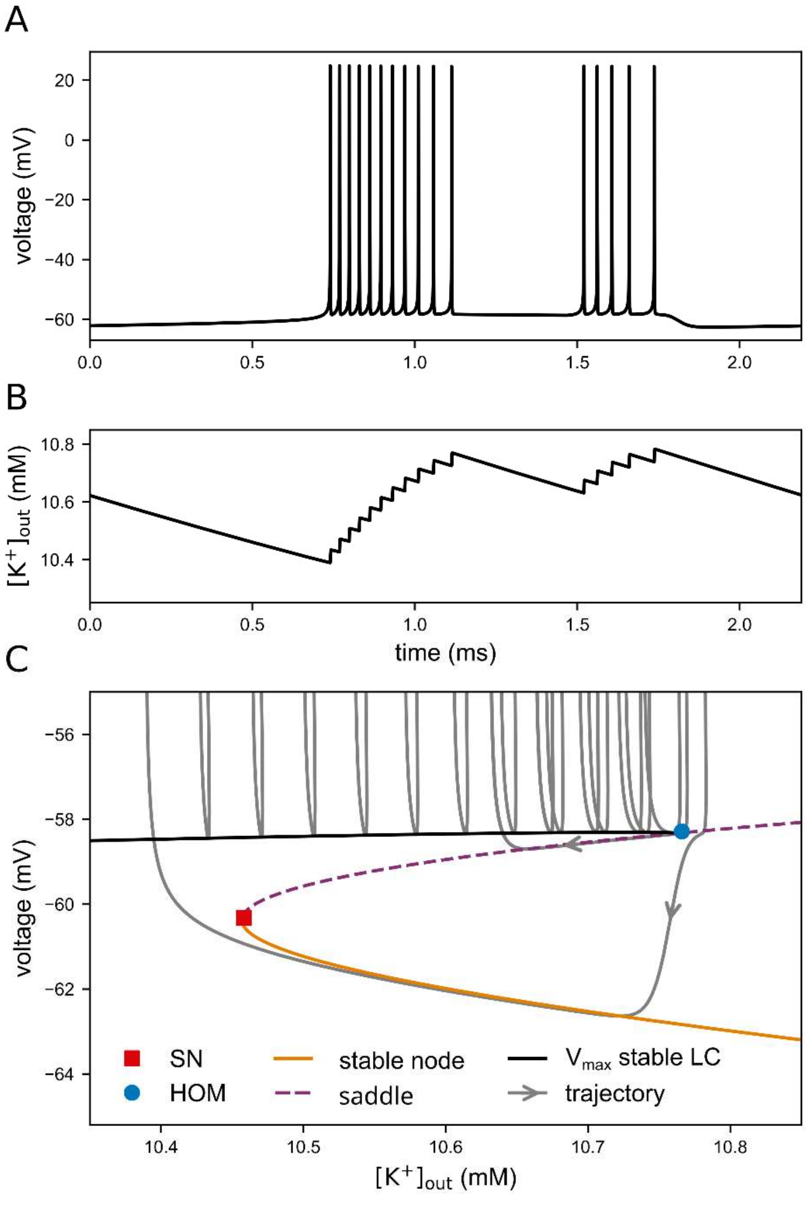

S2 Fig. Jump-on canard at the transition between 11-spike bursts and 12-spike bursts.

The spike-adding mechanism at play in our model appears to involve canard solutions. We show here a jump-on canard, found at the transition between the 11-spike burst that was used as illustrative example throughout this paper (Fig 2-4), and a 12-spike burst obtained when increasing the pump density I_{max}. Jump-on canards are particular solutions that connect a fast and attractive component (here when the neurons spikes), to a slow and repulsive component of the

dynamics (here the short pause in the spiking within the burst), see for example [85,86]. (**A**) Voltage trace during one period. (**B**) Potassium trace during one period. (**C**) Voltage trajectory superimposed onto the bifurcation diagram of the fast subsystem with respect to potassium. This trajectory follows for a while the repelling slow manifold defined by the saddle point of the fast subsystem involved in the homoclinic bifurcation (orange dashed line). I_{app}=0.5 μA/cm^2, I_{max}=0.99450852625 μA/cm^2.
