# Supplementary material for: The Na+/K+-ATPase generically enables deterministic bursting in class I neurons by shearing the spike-onset bifurcation structure
Source: PLoS Comput Biol. 2024 Aug 12;20(8):e1011751. doi: 10.1371/journal.pcbi.1011751 (PMC11383233; doi:10.1371/journal.pcbi.1011751)
Supplement: S3 Fig — The bursting mechanism introduced in this paper is not affected by the dependence of the Na+/K+-ATPase on [Na+]in. (A) Voltage and ionic concentration dynamics during bursting in a modified model with dynamic [Na+]in. Despite the addition of [Na+]in dynamics, the bursting described in this paper is still observed, with extracellular potassium accumulating during spiking and decreasing during rest, similar to the behaviour shown in Fig 2. (Iapp = 0.5 μA/cm2, Imax = 10 μA/cm2.) (B) Bursting dynamics for the modified model with fixed [Na+]in. By fixing [Na+]in at 18.4 mM (mean approximation from A) in the modified model, the bursting dynamic is the same as what is depicted in the main model of this paper (see Fig 2). (PDF) [file pcbi.1011751.s003.pdf]

S3 Fig for:

The Na^+/K^+-ATPase generically enables deterministic bursting in class I neurons by shearing the spike-onset bifurcation structure

Mahraz Behbood[1,2], Louisiane Lemaire[1,2], Jan-Hendrik Schleimer[1,2], Susanne Schreiber[1,2,*]

[1] Institute for Theoretical Biology, Department of Biology, Humboldt-Universität zu Berlin, Philippstraße 13, 10115 Berlin, Germany

[2] Bernstein Center for Computational Neuroscience, Philippstr. 13, 10115 Berlin, Germany

[*] Corresponding author E-mail: s.schreiber@hu-berlin.de (SS)

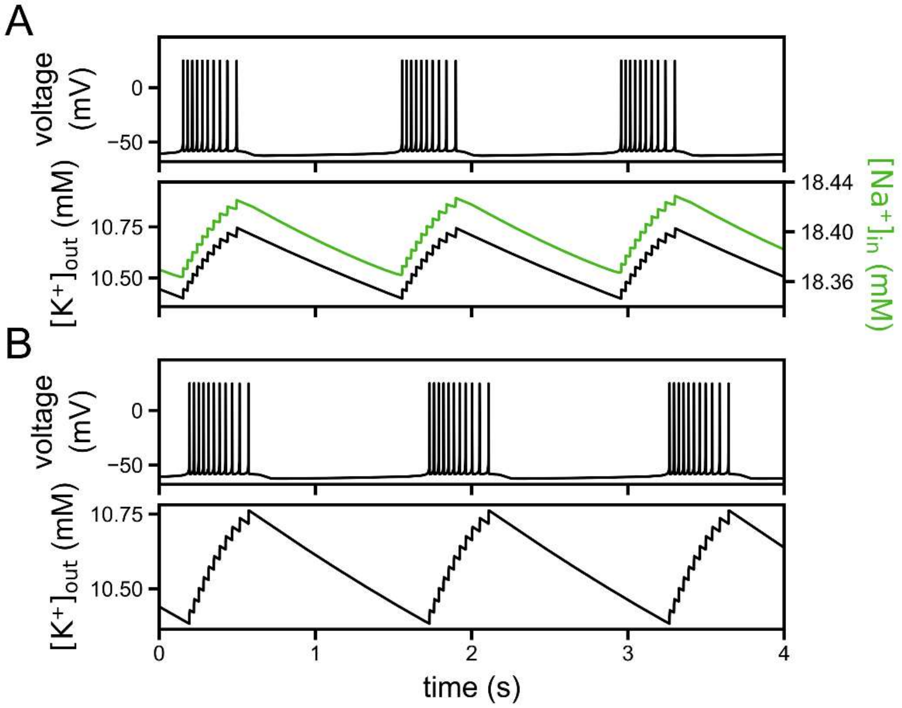

S3 Fig. Effect of intracellular sodium on bursting dynamic.

The bursting mechanism introduced in this paper is not affected by the dependence of the Na+/K+-ATPase on $[Na^+]_{in}$. (**A**) Voltage and ionic concentration dynamics during bursting in a modified model with dynamic $[Na^+]_{in}$. Despite the addition of $[Na^+]_{in}$ dynamics, the bursting described in this paper is still observed, with extracellular potassium accumulating during spiking and decreasing during rest, similar to the behaviour shown in Fig 2. (I_{app}=0.5 μA/cm^2, I_{max}=10 μA/cm^2.) (**B**) Bursting dynamics for the modified model with fixed $[Na^+]_{in}$. By fixing $[Na^+]_{in}$ at 18.4 mM (mean approximation from A) in the modified model, the bursting dynamic is the same as what is depicted in the main model of this paper (see Fig 2).
